# Addressing challenges in scaling up TB and HIV treatment integration in rural primary healthcare clinics in South Africa (SUTHI): a cluster randomized controlled trial protocol

**DOI:** 10.1186/s13012-017-0661-1

**Published:** 2017-11-13

**Authors:** Kogieleum Naidoo, Santhanalakshmi Gengiah, Nonhlanhla Yende-Zuma, Nesri Padayatchi, Pierre Barker, Andrew Nunn, Priashni Subrayen, Salim S. Abdool Karim

**Affiliations:** 1 Centre for the AIDS Programme of Research in South Africa (CAPRISA), Nelson R Mandela School of Medicine, Private Bag X7, Congella, Durban, 4013 South Africa; 2 CAPRISA-MRC TB-HIV Pathogenesis and Treatment Research Unit, Durban, South Africa; 3 Institute for Healthcare Improvement, Cambridge, MA USA; 4 Gillings School of Global Public Health, UNC Chapel Hill, Chapel Hill, United States of America; 5 Medical Research Council Clinical Trials Unit at University College London, London, UK; 6 BroadReach, Durban, South Africa; 7 Department of Epidemiology, Mailman School of Public Health, Columbia University, New York, NY USA

**Keywords:** Implementation science, TB-HIV co-infection, TB-HIV integration, Quality improvement

## Abstract

**Background:**

A large and compelling clinical evidence base has shown that integrated TB and HIV services leads to reduction in human immunodeficiency virus (HIV)- and tuberculosis (TB)-associated mortality and morbidity. Despite official policies and guidelines recommending TB and HIV care integration, its poor implementation has resulted in TB and HIV remaining the commonest causes of death in several countries in sub-Saharan Africa, including South Africa. This study aims to reduce mortality due to TB-HIV co-infection through a quality improvement strategy for scaling up of TB and HIV treatment integration in rural primary healthcare clinics in South Africa.

**Methods:**

The study is designed as an open-label cluster randomized controlled trial. Sixteen clinic supervisors who oversee 40 primary health care (PHC) clinics in two rural districts of KwaZulu-Natal, South Africa will be randomized to either the control group (provision of standard government guidance for TB-HIV integration) or the intervention group (provision of standard government guidance with active enhancement of TB-HIV care integration through a quality improvement approach). The primary outcome is all-cause mortality among TB-HIV patients. Secondary outcomes include time to antiretroviral therapy (ART) initiation among TB-HIV co-infected patients, as well as TB and HIV treatment outcomes at 12 months. In addition, factors that may affect the intervention, such as conditions in the clinic and staff availability, will be closely monitored and documented.

**Discussion:**

This study has the potential to address the gap between the establishment of TB-HIV care integration policies and guidelines and their implementation in the provision of integrated care in PHC clinics. If successful, an evidence-based intervention comprising change ideas, tools, and approaches for quality improvement could inform the future rapid scale up, implementation, and sustainability of improved TB-HIV integration across sub-Sahara Africa and other resource-constrained settings.

**Trial registration:**

Clinicaltrials.gov, NCT02654613. Registered 01 June 2015.

## Background

Tuberculosis (TB) is the commonest opportunistic infection and cause of death among human immunodeficiency virus (HIV)-infected patients in resource-limited countries [1]. In 2015, there were an estimated 10.4 million new (incident) TB cases worldwide, of which 5.9 million (56%) were among men, 3.5 million (34%) among women, and 1.0 million (10%) among children. In addition, people living with HIV accounted for 1.2 million (11%) of all the new TB cases [2]. In 2014, the World Health Organization (WHO) reported 83% incident TB cases worldwide out of which one third of these new TB cases originated from the African continent with high burden countries (HBCs) [3]. A similar trend was documented in the 2016 WHO global TB report which showed that the proportion of TB cases living with HIV was highest in the WHO African Region (31%) and exceeded 50% in parts of southern Africa [2].

The extent of the combined TB and HIV epidemics in South Africa has created enormous operational challenges for healthcare delivery [4]. Prior to the implementation of TB-HIV integration, the South African healthcare system provided separate vertical programmes for TB and HIV services delivered by different healthcare staff, often located in separate clinics [4–6]. The vertical model of delivering TB-HIV care to co-infected patients relied upon referral and linkage to care programmes (between TB and HIV programmes and vice versa). This proved problematic and inefficient as referral between programmes depended chiefly on patients’ resources and health-seeking behavior which was unmonitored [4, 6].

Integration according to Uyei et al. (2014) is operationalized by three domains: functional, organizational, and clinical integration [7]. An integrated model of TB and HIV healthcare service delivery is an efficient use of health system’s resources that would address the two very important co-epidemics [8]. A number of studies provide evidence of the relationships in the integration framework that applies to TB and HIV healthcare delivery [7, 9–11]. However, the optimum model for integrated TB-HIV services in a clinical setting is unknown.

### South African guidelines on TB-HIV integration

The South African Department of Health (SA DoH) has developed guidelines and policies supportive for the integration of TB-HIV services. The key focus areas for TB-HIV integration as standard of care in the most recently updated SA DoH guidelines [8, 12–14] is outlined in Table 1.

**Table 1:** Focused areas for TB-HIV integration

▪ Testing and counseling for HIV in all patients with TB. ▪ Intensified case finding for TB in HIV-infected patients. ▪ Isoniazid preventative therapy (IPT) for HIV-positive patients that screen TB negative. ▪ ART initiation for all TB-HIV co-infected patients. ▪ Cotrimoxazole therapy for TB-HIV co-infected patients. ▪ Enhanced retention in care strategies including the post-test counseling and use of community-based outreach workers. ▪ Enhanced ART and TB treatment adherence strategies including the use of community care workers for adherence support and community-based management of selected patients. ▪ A fully integrated data management system—adopting the approach of one patient, one appointment, one file, and one data management system.	

### Clinical benefit of known TB-HIV integration interventions

Randomized controlled trials have demonstrated that early initiation of ART during TB therapy improved survival of TB-HIV co-infected patients by 56% [15, 16]. Additionally, initiating ART early during TB treatment (within 2–4 weeks) increased AIDS-free survival by 34–68% among patients with advanced HIV disease [15, 17, 18]. In spite of TB-HIV integration being incorporated into international and South African guidelines, mortality rates in 2015 for TB-HIV co-infected patients in South Africa was 133 per 100,000 population, which is more than three times higher than mortality in HIV-negative TB patients, who had mortality rates of 46 per 100,000 population [2].

Initiation of cotrimoxazole preventive therapy (CPT) before or with ART, irrespective of CD4 count, in co-infected patients has been shown to reduce severe bacterial infections in an observational study [19] and mortality by 27% in a multi-site randomized clinical trial (RCT) conducted in Africa [20]. The clinical benefits associated with the use of CPT was adopted by WHO for use as an adjunctive therapy for improved outcomes in the management of TB and HIV co-infected patients in the 2014 treatment guidelines [20–22]. In addition, findings from a RCT conducted in South Africa showed that 12 months of isoniazid preventive therapy (IPT) conferred a 37% reduction in risk of active TB in ART-naïve patients [23]. The benefit of 6 months of IPT and early ART irrespective of baseline CD4 count was also recently confirmed in the TEMPRANO study that showed 44% lower risk of severe HIV-related illness and a 35% lower risk of death from any cause [24].

### Rationale of the study

Despite the inclusion of the evidence above in standard TB and HIV treatment guidelines, implementation of these interventions as part of TB-HIV integration in South Africa remains poor [23, 25, 26]. Premature mortality from TB and HIV in women and men account for 53.7 and 24.2% of deaths in the 15–24 year age group and for 44.4 and 47.2% of deaths in the 25–64 age group, respectively [27]. Cost-effective and sustainable strategies to strengthen integration of known effective TB-HIV interventions in primary health care (PHC) clinics, the main service delivery point for millions of patients, will abrogate the high mortality associated with TB-HIV co-infection [28]. A systematic review by Uyei and colleagues in 2011 of observational studies using secondary data on TB-HIV integration in sub-Saharan Africa found several benefits from integration and identified the need for additional research to identify barriers to integration as well as strategies to improve TB-HIV integration [26].

### Theoretical framework

Models of TB-HIV integration have ranged from TB clinics referring patients to HIV clinics and vice versa to full integration where both services are available at a single facility, on the same day, by the same healthcare worker [5]. A group of South African researchers developed a model to illustrate the critical health system mechanisms that are essential to operationalize TB and HIV service integration [7]. Implementing integration requires *Functional integration* which is the extent to which integration is supported at the policy and budget level; *Clinical integration* which is the extent to which TB and HIV care, treatment, diagnostic testing, and health education activities are taking place concurrently; and *Organizational integration* which is the extent to which facility level resources (e.g., staff, infrastructure, space, patient files, and data systems) and processes (e.g., patient flow) are integrated [7]. While the model efficiently explains *what* is needed to integrate TB and HIV services, it does not illustrate *how* TB and HIV service integration should be integrated. Quality improvement as a method to improve organizational and clinical integration is on the cusp of widespread roll out in South African Department of Health facilities and there is enough support and political will for creating a culture of quality improvement (QI) in facilities [8, 14, 29].

**Fig. 1:**
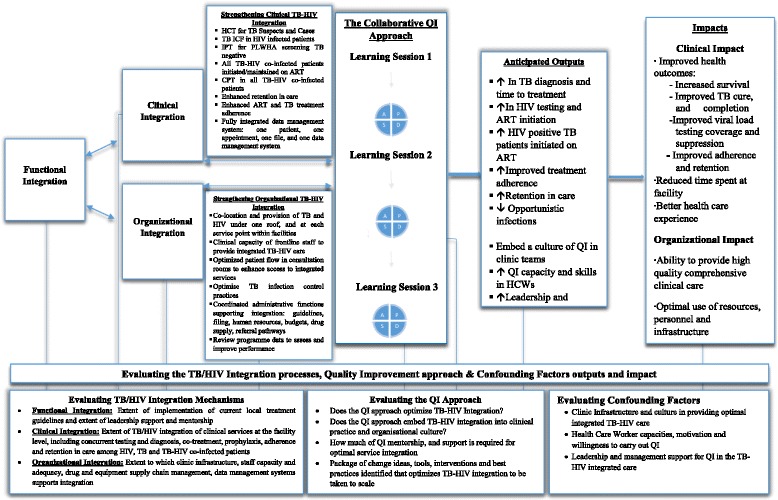
SUTHI study theoretical framework. IPT isoniazid preventive therapy, CPT cotrimoxazole preventive therapy, PLWHA people living with HIV/AIDS, ICF intensified case finding, HCT HIV counseling and testing

We propose a theoretical framework which is an adaptation of the Uyei et al. (2014) model that demonstrates the central role of QI as a catalyst to operationalizing integration. The SUTHI study intervention has targeted PHC supervisors and frontline clinic staff as the recipients of the QI intervention. Figure 1 illustrates that through continuous QI activities, we theorize that organizational and clinical integration can be improved and strengthened and lead to improved patient and organizational outcomes. Using the collaborative breakthrough series approach, developed by the Institute of Healthcare Improvement (IHI) [30–33], we propose that a series of timed collaborative learning sessions that brings intervention clinics together coupled with mentorship visits at the facility level will impact clinical and organizational integration activities. Our framework proposes that QI alone may not be sufficient to bring about improvements in integrated service delivery hence evaluating, measuring, and monitoring environmental and contextual factors (confounders) as well as delivering a good quality QI intervention is key to improving TB, HIV, and integrated TB-HIV services.

## Methods

### Aims and objectives

This study aims to reduce mortality due to TB, HIV, and TB-HIV co-infection through a QI strategy for scaling up of TB and HIV treatment integration in rural PHC clinics in South Africa. The hypothesis is that survival rates will be lower in TB, HIV, and TB-HIV co-infected patients accessing health care at clinics implementing the study intervention to deliver integrated TB-HIV care, compared to survival in patients accessing health care at clinics that provide only the standard of care for people with TB and or HIV.

Specific objectives include:

1. To determine the impact of a QI-mediated TB-HIV care integration on patient mortality.
2. To determine the effectiveness of peer-led QI approach to enhance integration of TB-HIV healthcare delivery.
3. To identify clinic-level factors that impact on the implementation of integrated TB-HIV services.
4. To determine the cost-effectiveness of implementing TB-HIV integration using the QI approach.
5. To develop an intervention, comprising QI-based change ideas, tools, and approaches for the scale up, implementation, and sustainability of integrated TB-HIV services across South Africa and in other resource-constrained settings.

### Study setting and design

This is an open-label cluster randomized controlled trial where the cluster is defined as the group of clinic(s) under the same PHC clinic supervisor, where each of the 16 PHC clinic supervisors may oversee between 3 and 5 PHC clinics. Cluster randomization was chosen for practical reasons because the study will be carried out in pragmatic settings involving 40 PHC clinics within the King Cetshwayo district (formerly called uThungulu) [34] and Ugu districts in KwaZulu-Natal, South Africa. A total of 11.1 million people (19.9% of the South African population) live in KwaZulu-Natal [35]. The province has the highest TB-HIV disease burden in South Africa with an estimated TB-HIV co-infection rate of 70% [12, 36]. The two districts were selected because they are rural with high burden of TB, HIV, and TB-HIV co-infection, despite TB and HIV treatment services in accordance with current guidelines being available [13, 14, 37]. In 2016, 82.3% of TB-HIV co-infected patients were initiated on ART in KwaZulu-Natal, slightly lower than the national ART initiation rate of 84.5% [38]. King Cetshwayo district, located on the northern coast of KwaZulu-Natal, has a population of 937,793, with approximately 80% living in rural settings [39]. Similarly, Ugu district, situated in southern KwaZulu-Natal has 86% of its population of 750,214 living in rural areas [38, 40]. The in-hospital case fatality rate due to TB-HIV co-infection in both King Cetshwayo district (38.4%) and Ugu district (38.8%) remains high despite ART availability [27]. In 2015, over a quarter of all deaths in KwaZulu-Natal, irrespective of age or gender, were caused by TB (15.5%) or HIV (12.2%). PHC clinics offer community level frontline services and chronic care for all health ailments, including TB and HIV. Patient level data for these diseases are routinely captured electronically [41].

### Study population

Anonymized clinical and programme outcome data for all TB, HIV, and TB-HIV co-infected patients accessing services in the 40 clinics will be included in the study analysis. According to estimation from the clinic headcount data, an average of 4500 patients are seen in the two districts each month. In addition, the healthcare workers (HCWs) including PHC supervisors will be interviewed to collect clinic level information after obtaining informed consent for participation in interviews. These interviews will be collated and analyzed together with patient level data.

### Randomization and study groups

**Fig. 2:**
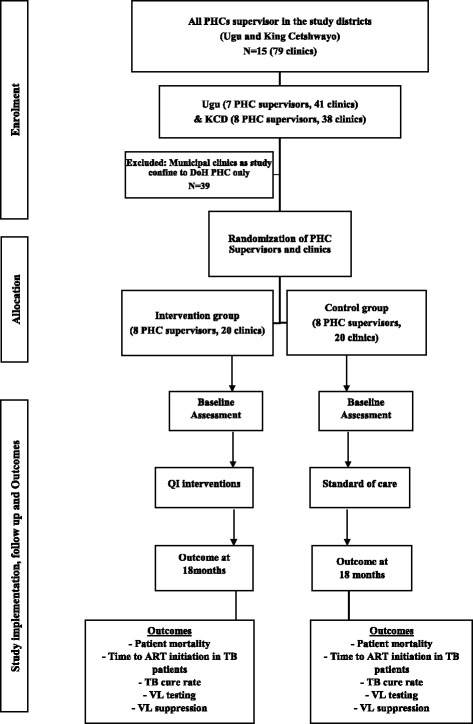
SUTHI study enrolment, randomization, follow-up, and outcome following TB-HIV integration intervention

Randomization will occur at the level of the PHC supervisor. This is being done to avoid contamination of intervention effects into control clinics since each supervisor routinely supports multiple clinics. Each PHC clinic supervisor will be randomly assigned in a 1:1 ratio to the intervention or control arm. The study investigators would provide a list of facilities and supervisors to be randomized using a computer-generated randomization list generated by study statistician. In South Africa, a PHC clinic supervisor fulfills a management role at PHC clinics [42], and are mandated to ensure smooth clinic operations through oversight of clinic performance against set targets including patient clinical outcomes and programme performance targets, and oversees the implementation of clinical guidelines. Each PHC clinic supervisor may oversee between three and five PHC clinics within one district. Therefore, through randomization of 14–16 PHC clinic supervisors working in both the Ugu district and King Cetshwayo district, 40 clinics (20 in each arm) will be enrolled into this study as each supervisor oversees on average three to five medium-sized clinics. Not all clinics under the purview of the PHC clinic supervisors randomized will be enrolled into the study. Clinics will be excluded if ART services are not offered on site, services are run by a single nurse, and if they are mobile clinics or clinics located within hospitals. All eligible clinics enrolled in the control arm of this trial will receive the prevailing standard-of-care for TB-HIV integration (Fig. 2) in accordance with the DoH guidelines. While the study team will not interfere with any additional benefit that control clinics may receive (for example, support from other NGOs), changes to the background standard of care will be recorded systematically and accounted for in the final analysis.

### The quality improvement intervention

Quality improvement is described as a set of continuous actions through coordinated effort of HCW teams aimed at accelerating improvements to patient care and health outcomes [43]. This is done through iterative changes and peer-to-peer learning about successful changes and has been demonstrated to significantly improve HCW performance and lead to sustainable delivery of quality care with improved health outcomes [30, 43, 44]. The Institute for Healthcare Improvement (IHI) Breakthrough Series (BTS) is an example of quality improvement through learning collaborative, demonstrated to successfully impact health outcomes in developing-country settings [30, 45]. The learning collaborative brings together clinic teams at regular intervals to learn QI methods and exchange challenges and successes in efforts to implement the TB-HIV care pathway (learning sessions). Each clinic will form a QI team that will work with the PHC supervisor and QI mentors between these learning sessions to develop local ideas for implementing a specific area of care, regularly measure performance using agreed-upon indicators, and bring back those results and emerging best practices to other clinic teams through periodic learning sessions. We aim to use this structured implementation approach to improve reliable delivery of published evidence-based interventions known to decrease morbidity and mortality in patients with TB and HIV. The main measure of the collaborative’s success will be measurable improvement in the magnitude, maintenance, and speed of specific steps of TB-HIV integration, ascertained through time-series analyses. These analyses will be collected and acted upon in real-time and will include process and outcome indicators. The magnitude of TB-HIV integration implementation will be measured by the extent to which interventions provide measurable improvements in PHC process indicators from baseline (e.g., coverage of HCT and IPT services among eligible patients, improvements in quality of services, proportion of HIV-infected patients offered IPT following a negative TB screening). We will also track the speed at which each of the eight TB-HIV integration interventions are implemented (e.g., the number of months taken to reach 90% implementation of individual integration interventions) and factors affecting speed of intervention uptake in the clinics.

Systematic testing of change ideas will be evaluated through a rapid sequence of steps called the Plan-Do-Study-Act (PDSA) cycle. The PDSA cycle is a sequential framework for examining problems, deriving solutions, measuring progress, and embedding changes leading to positive outcomes [46, 47]. Selected HCWs from the study intervention clinics will be trained on QI methodology. This training will enable establishment of QI teams in intervention clinics to work test and implement change ideas to advance implementation of the TB-HIV integration interventions in rapid cycle (Fig. 1) and exchange successful ideas for change with other intervention sites through the learning collaborative. Furthermore, measures will be taken to prevent inadvertent cross-contamination of change ideas (e.g., by avoiding any convening of intervention and control clinics or personnel).

### Training, coaching, and mentorship for HCWs implementing QI in clinics randomized to the intervention arm

The QI approach promotes front-line staff engagement in the identification of problems affecting performance and catalyzing rapid cycle testing of possible solutions in each of the eight identified TB-HIV integration indicators (Table 1). The QI team at each of the intervention clinics will include the PHC supervisor from the District Health Management team, the QI clinic champion, the clinic’s operation manager, selected clinic staff members, and a QI coach/mentor represented by a member of the study team. The QI champion, usually a clinic’s most senior nursing staff, will be trained to lead and support QI teams in their facilities using QI methods including the PDSA model, systems thinking, and the use of data for improvement.

The QI mentor and champion will be capacitated by the research team to provide peer-leadership and mentorship for implementation of QI methodology. In addition, they will also be capacitated to monitor the weekly performance of their clinics in achieving key successes on the indicators being targeted for improvement. The QI teams will be established through training of selected health workers to become fully fledged QI leaders and implementers within their facilities. Only HCWs experienced in both TB-HIV management and implementation of national TB and HIV guidelines would be eligible for QI training.

### Implementation of the intervention

Three collaborative learning sessions and additional QI support (mentoring and coaching) visits at specific time points during the study period (Fig. 1) will be pre-arranged. Collaborative learning session one will coach teams in the use of a range of QI methods and tools (process mapping, fishbone system analysis, PDSA cycles, use of line graphs, and other data for improvement). Areas requiring improvement will be identified and prioritized, and aim statements encompassing specific aims and targets will be developed. Brainstorming by the QI team and process mapping will be done to define a strategy to effect positive change (“a change idea”). The PDSA cycles tracking a set of predefined indicators will be reviewed bi-weekly by the QI team to test if the recently adopted change idea resulted in performance improvement, throughout the study period. This will enable the generation of new ideas for improvement, iterative testing of these ideas, and monitoring of progress attained through use of run charts and graphs. Learning session two, scheduled 6 months later will review learnings for all successful change ideas and challenges against overall clinic performance, with the goal of scaling back on frequency of QI support meetings for clinics that are undertaking reliable improvement work. Learning session three, scheduled at month 12, will be focused on review and scalability of the final successful change package. It is anticipated that after learning session three, the clinic’s QI teams would sustain successful change ideas. Lessons learned will also be shared and documented across the intervention teams. At the successful conclusion of the projects, the intention is to scale up a reliable “change package” of successful, tested changes across all clinics in the two districts, if not across the entire district.

### Standard of care in the control clinics: description of TB, HIV, and integration services

Since the 2009 endorsement of integrated TB-HIV services by the South African National AIDS Council (SANAC) [48], and adoption of TB-HIV service integration into policy and practice by the National Department of Health (NDoH) [29], South African guidelines recommend integration of TB and HIV services by a single service provider [8, 49].

### Data collection

Data on TB-HIV integration indicators will be obtained from patient files and existing standard customized electronic data management systems supported by SA DoH [4, 41]. Routine clinical information is recorded in paper-based registers and patient clinic folders housed in clinics and subsequently captured onto the relevant TB or HIV electronic system supported by SA DoH. Baseline patient level data will be collected retrospectively for the 12 months prior to study start and will continue prospectively throughout the study period (Tables 2 and 3). In addition, HCW interviews will be conducted, using case report forms, at specific time points during study period from consenting health workers in both the intervention and control clinics. Table 2 summarizes the data to be collected, data source, and outcome measures while Table 3 presents the study’s schedule of activities.

**Table 2:** Data collection tools, sources, and outcome measures

Data to be collected	Data source	Outcomes measure
TB-HIV integration indicators		Clinical outcomes
	TIER.Net, community care givers, autopsy reports	- Mortality rates—number of deaths among TB and HIV patients accessing care in study clinics from date clinic enrolled to 18 months post enrolment.
	TIER.Net	- Proportion of patients retained in care—proportion of HIV-infected patients enrolled in care at clinics and alive 12 months.
	TIER.Net	- Viral load testing coverage—proportion of patients on ART with viral loads test done among those eligible for viral load test at requested time points.
	TIER.Net	- Viral load suppression—proportion of patients with undetectable viral load tests among those receiving 12 monthly viral load test.
	TIER.Net and clinic TB registers	- TB treatment outcomes at end of study period— - Cure rates: proportion of new smear-positive patients that are smear-negative in the last month of treatment and on at least one other occasion at least 30 days prior. - Loss to follow-up rates: proportion of new smear-positive patients that interrupted TB treatment for 2 consecutive months or more. - Treatment failure rates: Proportion of new smear-positive patients that are smear-positive at the end of TB treatment period. - Death rate: proportion of new smear-positive patients that died during TB treatment. - Transfer-out rate: proportion of new smear-positive pulmonary TB patients registered that were transferred to another district and for whom the TB treatment outcome is unknown.
		*Process outcome* - HCT Coverage—proportion of patients with unknown HIV status tested for HIV
	TIER.Net, DHIS and clinic-based registers	
	TIER.Net, DHIS and clinic-based registers	- Co-infection—proportion of TB patients co-infected with HIV
	TIER.Net, DHIS and clinic-based registers	- Time to ART initiation (in days)—time in days between diagnosis of HIV infection diagnosis and ART initiation.
	TIER.Net, DHIS and clinic-based registers	- TB screening coverage among HIV-infected patients— a) Proportion of HIV-infected patients receiving TB screening and b) Frequency of TB screening during follow-up
	TIER.Net, DHIS and clinic-based registers	- IPT initiation— a) Proportion of HIV-infected TB negative patients initiated on IPT and b) Proportion of patients completing IPT course.
	TIER.Net, DHIS and clinic-based registers	- CPT uptake among co-infected patients—proportion of eligible HIV-positive patients initiated on CPT
TB-HIV service integration in the facility macro-environment	Survey instrument developed by Uyei et al. 2014 [7]	Measured TB-HIV integration in terms of: ➢ Organization—such as co-location of services, combined patient records, information management, and joint training ➢ Structure—existent practice of joint service delivery, ➢ Process—behavior and practice of delivering services ➢ Culture—work place culture and personal identification with integrated service delivery
Clinic profile tool aimed at assessing clinics’ infrastructure, capacity, and systems in place to implement TB-HIV integration services	A CAPRISA designed tool	- Resources inventory and needs for implementation of TB-HIV integration services, e.g., available guidelines, protocols, policies, trained staff. - Existing quality improvement interventions, processes and measurements - District level leadership and support
Clinic culture, leadership, resources, etc.	The COACH tool designed by Bergstrom et al. 2015 [56]	- Clinic leadership and support - Staff knowledge and skills - Perceptions of work culture at PHC
Staff Work-related Quality of Life	WHO Work-related Quality of Life Scale	Work-related quality of life for staff at PHC

**Table 3:** Study schedule of activities

Study activity	Study time points			
	Baseline (0–1 month)	6–7th month	12–13th month	Monthly (1st–18th month)
Retrospective collection of 12 months data on TB-HIV indicators from TIER.Net*, DHIS** and clinic-based registers	X			
***QI Learning Collaborative (Intervention Clinics Only)	X	X	X	
Monthly downloads of data on TB-HIV indicators from TIER.Net*, DHIS** and clinic-based registers				X
Clinic Profile Survey	X	X	X	
TB/HIV Service Integration Survey	X	X	X	
Work-related Quality of Life Survey	X	X	X	
Context Assessment Survey	X	X	X	
Quality Improvement Survey (Intervention Clinics Only)	X	X	X	

*TIER.Net—Three Inter-Linked Electronic Register for Tuberculosis

**DHIS—District Health Information System

***QI Learning Collaborative—use of PDSA cycles, run charts, process mapping

### Study outcome measures

The primary outcome of this study will be all-cause mortality rate among patients newly diagnosed with TB and/or HIV. Secondary outcomes will include time to ART initiation, retention in care, IPT and CPT uptake as per the current SA DoH guidelines, TB cure rates, viral load testing rates, and viral load suppression rates.

### Sample size estimation

We anticipate that about 6000 HIV-positive patients will be diagnosed with active TB in the study population during a 12-month period. This is based on the assumption that between 100 and 200 new TB-HIV co-infected patients are seen in each of the 40 clinics per year. This translates to an average of 350 patients per cluster (assume unequal cluster sizes). Assuming a case fatality rate of 15% in the control arm, coefficient of variation between 0.25 and 0.35, and a type I error rate of 5%, we will have 80% power to detect a 30% reduction in mortality. The chances of detecting other levels of effectiveness are shown in Table 4.

**Table 4:** Power to detect different levels of effectiveness (keeping number of events constant)

Reduction in mortality (%)	Power to detect an effect (%)
10	13
20	42
30	80
40	98

### Data management

Anonymized TB, HIV, and TB-HIV co-infected patient data obtained from electronic software systems supported by the DoH and clinic-based registers will be collated for quality control and for evaluation of impact made by the intervention in data quality. Also, iDataFax version 2014.1.1 which is clinical data management software will be used for the design of case reporting forms for the HCW interviews and its data entry into the study’s database. Both intervention and control clinics will have data mentoring by the study data management team to ensure that quality of data obtained are improved, standardized, reliable, and valid. The study database files will be password-protected, and access to the files will be limited to authorized study staff members. Quality control measure will be carried out periodically throughout the study period prior to the data analysis.

### Statistical analysis

The primary outcome of mortality and secondary outcomes will be analyzed using cluster-summary methods. The primary outcome will be analyzed among TB-HIV co-infected patients only while the secondary outcomes will be analyzed among TB-HIV co-infected, HIV-only, and TB-only patients. Mortality rate per arm will be calculated as geometric mean of cluster-level summaries and will be compared using unpaired *t* test. The same technique will be applied to secondary outcomes. The *t* test applied in cluster-level summaries is one of the robust methods of analyzing unmatched trials especially when there are small number of clusters per arm [50]. Since we have 16 clusters and 40 sub-clusters, multi-level regression will be used as secondary analyses. Proportional hazard regression with random effects (frailty models) will be used for analyses of time-to-event outcomes. Generalized estimating equations and mixed effects linear models will be used for binary and quantitative outcomes, respectively. These models will take the clustering by PHC supervisor and clinic into account through the random effects. They will also allow us to adjust for baseline variables, especially those with imbalance between arms, as this is likely when clusters are fewer. The HCW interviews will be summarized using descriptive statistics such as means and frequencies. Adjusted baseline descriptive statistics will be calculated as the means of the cluster-level summaries and characteristics of the two arms will be compared using *t* test or rank sum tests. Individual-level summaries at baseline will be compared using *t* test or rank sum tests and Fisher’s exact test. Data will be analyzed with SAS version 9.4 (or higher) (SAS Institute INC., Cary N.C, USA).

## Discussion

Findings from this trial are expected to provide information on a scalable strategy (a “change package”) to address shortcomings in the implementation of integrated TB-HIV treatment and services. If successful, the strategy could make a contribution to reducing TB-HIV-associated mortality and morbidity in South Africa and other regions of the world where co-infection with TB and HIV is a concern. In 2013, WHO performed a joint review of HIV, TB, and prevention of mother to child transmission (PMTCT) programmes in South Africa, which recommended the need for context-specific mechanisms for the delivery of integrated TB-HIV services at PHC and community level, with particular focus on improving access to TB and HIV services for children, adolescents, and key populations. Some studies undertaken in the South African PHC clinic setting have shown that the QI strategy can be effective as an intervention for PMTCT care [51–54]. To date, there have been no studies that have explored the use of the QI model as an intervention to improve integration of TB and HIV treatment. An important component of the QI model is the PDSA cycle, comprising learning cycles to test and revise theory-based predictions as recommended by Taylor et al. (2014) in their systematic review on the application of the PDSA method to improve quality in healthcare [55]. We anticipate that the findings from this trial will offer an affordable and sustainable strategy through use of the QI model to effectively improve the integration of TB-HIV programmes. The study results will be communicated to stakeholders through dissemination meetings, conferences, and publication in peer-reviewed journals. Recommendations would be made based on the study findings for appropriate actions to be considered and taken by the department of health and relevant authorities in areas with high burden of TB and HIV.

### Trial status

Data collection is currently on-going.

## Abbreviations

ART: Antiretroviral therapy
BREC: Biomedical research ethics committee
CAPRISA: Centre for the AIDS programme of research in South Africa
COACH: Context assessment for community health
CPT: Cotrimoxazole preventive therapy
DHIS: District Health Information System
DoH: Department of Health
GEE: Generalized estimation equations
HBC: High burden countries
HCT: HIV counseling and testing
HCWs: Healthcare workers
HIV: Human immunodeficiency virus
IPT: Isoniazid preventive therapy
PDSA: Plan-Do-Study-Act
PHC: Primary health care
PMTCT: Prevention of mother to child transmission
QI: Quality improvement
SA: South Africa
SUTHI: Addressing challenges in scaling up TB and HIV treatment integration in public health settings in South Africa
TB: Tuberculosis
TIER.Net: Three interlinked electronic register for tuberculosis and human immunodeficiency virus
WHO: World Health Organization

## References

[1] Manosuthi W, Wiboonchutikul S, Sungkanuparph S (2016). Integrated therapy for HIV and tuberculosis. AIDS Res Ther.

[2] World Health Organization. Global Tuberculosis Report 2016. Available from http://apps.who.int/medicinedocs/documents/s23098en/s23098en.pdf. Accessed 12 Feb 2017.

[3] World Health Organization. Global Tuberculosis Report 2015. Available from http://www.who.int/tb/publications/global_report/en/. Accessed 28 Aug 2016.

[4] Kaplan R, Caldwell J, Bekker LG, Jennings K, Lombard C, Enarson DA (2014). Integration of TB and ART services fails to improve TB treatment outcomes: comparison of ART/TB primary healthcare services in Cape Town, South Africa. South African medical journal = Suid-Afrikaanse tydskrif vir geneeskunde.

[5] Legido-Quigley H, Montgomery CM, Khan P, Atun R, Fakoya A, Getahun H (2013). Integrating tuberculosis and HIV services in low- and middle-income countries: a systematic review. Tropical medicine & international health: TM & IH.

[6] Daftary A, Padayatchi N (2013). Integrating patients’ perspectives into integrated tuberculosis-human immunodeficiency virus health care. The international journal of tuberculosis and lung disease : the official journal of the International Union against Tuberculosis and Lung Disease.

[7] Uyei J, Coetzee D, Macinko J, Weinberg SL, Guttmacher S (2014). Measuring the degree of integrated tuberculosis and HIV service delivery in Cape Town. South Africa Health Policy Plan.

[8] McCarthy K, Goemaere E, Wilkinson L, Tihon V, Vilakazi-Nhlapo K, Hausler H,. A practical guide for TB and HIV service integration at primary health care facilities 2011. Available from: http://www.inpracticeafrica.com/~/media/Guidelines/Practical_Guide_TBHIV.pdf.

[9] Tweya H, Feldacker C, Gadabu OJ, Ng'ambi W, Mumba SL, Phiri D (2016). Developing a point-of-care electronic medical record system for TB/HIV co-infected patients: experiences from Lighthouse Trust, Lilongwe, Malawi. BMC Res Notes.

[10] Charles MK, Lindegren ML, Wester CW, Blevins M, Sterling TR, Dung NT (2016). Implementation of tuberculosis intensive case finding, isoniazid preventive therapy, and infection control (“three I’s”) and HIV-tuberculosis service integration in lower income countries. PLoS One.

[11] Webb Mazinyo E, Kim L, Masuku S, Lancaster JL, Odendaal R, Uys M (2016). Adherence to concurrent tuberculosis treatment and antiretroviral treatment among co-infected persons in South Africa, 2008-2010. PLoS One.

[12] Department of Health Province of Kwazulu-Natal (2015). Strategic plan 2015–2019.

[13] Department of Health Republic of South Africa. National Tuberculosis Management Guidelines. Pretoria: National Department of Health; 2014. Available from: http://www.nicd.ac.za/assets/files/National TB management guidelines 2014.pdf. Accessed 18 Feb 2015.

[14] National Department of Health (2016). Adherence guidelines for HIV, TB and NCDS.

[15] Abdool Karim SS, Naidoo K, Grobler A, Padayatchi N, Baxter C, Gray AL (2011). Integration of antiretroviral therapy with tuberculosis treatment. N Engl J Med.

[16] Uthman OA, Okwundu C, Gbenga K, Volmink J, Dowdy D, Zumla A (2015). Optimal timing of antiretroviral therapy initiation for HIV-infected adults with newly diagnosed pulmonary tuberculosis: a systematic review and meta-analysis. Ann Intern Med.

[17] Blanc FX, Sok T, Laureillard D, Borand L, Rekacewicz C, Nerrienet E (2011). Earlier versus later start of antiretroviral therapy in HIV-infected adults with tuberculosis. N Engl J Med.

[18] Havlir DV, Kendall MA, Ive P, Kumwenda J, Swindells S, Qasba SS (2011). Timing of antiretroviral therapy for HIV-1 infection and tuberculosis. N Engl J Med.

[19] Okwera A, Mafigiri DK, Guwatudde D, Whalen C, Joloba M (2015). Level of understanding of co-trimoxazole use among HIV infected, recurrent pulmonary tuberculosis suspects at a national referral tuberculosis clinic in Kampala, Uganda: a qualitative analysis. Afr Health Sci.

[20] Walker AS, Ford D, Gilks CF, Munderi P, Ssali F, Reid A (2010). Daily co-trimoxazole prophylaxis in severely immunosuppressed HIV-infected adults in Africa started on combination antiretroviral therapy: an observational analysis of the DART cohort. Lancet (London, England).

[21] Harries AD, Lawn SD, Suthar AB, Granich R (2015). Benefits of combined preventive therapy with co-trimoxazole and isoniazid in adults living with HIV: time to consider a fixed-dose, single tablet coformulation. Lancet Infect Dis.

[22] Suthar AB, Vitoria MA, Nagata JM, Anglaret X, Mbori-Ngacha D, Sued O (2015). Co-trimoxazole prophylaxis in adults, including pregnant women, with HIV: a systematic review and meta-analysis. The Lancet HIV.

[23] Churchyard GJ, Mametja LD, Mvusi L, Ndjeka N, Hesseling AC, Reid A (2014). Tuberculosis control in South Africa: successes, challenges and recommendations. S Afr Med J.

[24] Danel C, Moh R, Gabillard D, Badje A, Le Carrou J, Ouassa T (2015). A trial of early antiretrovirals and isoniazid preventive therapy in Africa. N Engl J Med.

[25] Nglazi MD, Kaplan R, Caldwell J, Peton N, Lawn SD, Wood R (2012). Antiretroviral treatment uptake in patients with HIV-associated TB attending co-located TB and ART services. S Afr Med J.

[26] Uyei J, Coetzee D, Macinko J, Guttmacher S (2011). Integrated delivery of HIV and tuberculosis services in sub-Saharan Africa: a systematic review. Lancet Infect Dis.

[27] Massyn N, Peer N, Padarath A, Barron P, Day C. District health barometer 2014/15. Health Systems Trust: Durban; 2015. Available from: http://www.hst.org.za/publications/District%20Health%20Barometers/Complete_DHB_2014_15_linked.pdf.

[28] Naidoo K, Baxter C, Abdool Karim SS (2013). When to start antiretroviral therapy during tuberculosis treatment?. Curr Opin Infect Dis.

[29] South African National AIDS Council. Progress Report on the National Strategic Plan for HIV, TB AND STIs (2012 – 2016). Pretoria: South African National AIDS Council. 2014. Available from https://www.healthe.org.za/wp-content/uploads/2014/12/SANAC-NSP-Progress-Report-2014.pdf.

[30] Franco LM, Marquez L (2011). Effectiveness of collaborative improvement: evidence from 27 applications in 12 less-developed and middle-income countries. BMJ quality & safety..

[31] Schouten LM, Hulscher ME, van Everdingen JJ, Huijsman R, Grol RP (2008). Evidence for the impact of quality improvement collaboratives: systematic review. BMJ (Clinical research ed).

[32] Scoville R, Little K, Rakover J, Luther K, Mate K. Sustaining Improvement. IHI White Paper. Cambridge: Institute for Healthcare Improvement; 2016. Available from ihi.org.

[33] Wyatt R, Laderman M, Botwinick L, Mate K, Whittington J. Achieving Health Equity: A Guide for Health Care Organizations. IHI White Paper. Cambridge: Institute for Healthcare Improvement; 2016. Available from: ihi.org.

[34] Khoza A (2016). Zwelithini welcomes renaming of uThungulu to King Cetshwayo District Municipality.

[35] Statistics South Africa (2016). Mid-year population estimates 2016. Stat. SA, Pretoria.

[36] Department of Health Province of Kwazulu-Natal (2015). Annual Report.

[37] National Department of Health. National consolidated guidelines for the prevention of mother-to-child transmission of HIV (PMTCT) and the management of HIV in children, adolescents and adults. Pretoria: Department of Health; 2015. Available from: http://www.sahivsoc.org/Files/ART Guidelines 15052015.pdf. Accessed 24 June 2015.

[38] Massyn N, Peer N, English R, Padarath A, Barron P, Day C. District health barometer 2015/16. Health System Trust: Durban; 2016. Available from: http://www.hst.org.za/publications/District%20Health%20Barometers/Complete_DHB_2015_16_linked.pdf

[39] KwaZulu-Natal Department of Health (2016). District health plan 2015/2016- uThungulu district.

[40] KwaZulu-Natal Department of Health (2016). District health plan 2015/2016- Ugu district.

[41] Osler M, Hilderbrand K, Hennessey C, Arendse J, Goemaere E, Ford N (2014). A three-tier framework for monitoring antiretroviral therapy in high HIV burden settings. J Int AIDS Soc.

[42] South African National Department of Health. Primary Health Care Supervision Manual 2009. Available from https://www.medbox.org/za-community-health/primary-health-care-supervision-manual-a-guide-to-primary-health-care-facility-supervision/preview? Accesed 22 Mar 2017.

[43] Batalden PB, Davidoff F (2007). What is “quality improvement” and how can it transform healthcare?. Quality & safety in health care.

[44] United State Department of Health and Human Services Health Resources and Services Administration. Quality improvement. 2011. http://www.hrsa.gov/quality/toolbox/508pdfs/qualityimprovement.pdf. Accessed 14 Sept 2016.

[45] Rowe A, Rowe SY, Peters DH, Holloway KA, Chalker J, Ross-Degnan D, Peters DH, El-Saharty S, Siadat B (2009). Review of strategies to improve health care provider performance. Improving health service delivery in developing countries. From evidence to action.

[46] Langley GLMR, Nolan KM, Nolan TW, Norman CL, Provost LP (2009). The improvement guide: a practical approach to enhancing organizational performance.

[47] Colbourn T, Nambiar B, Bondo A, Makwenda C, Tsetekani E, Makonda-Ridley A (2013). Effects of quality improvement in health facilities and community mobilization through women's groups on maternal, neonatal and perinatal mortality in three districts of Malawi: MaiKhanda, a cluster randomized controlled effectiveness trial. Int Health.

[48] National Department of Health (2014). Joint review of HIV, TB and PMTCT Programmes in South Africa.

[49] KwaZulu-Natal Department of Health (2016). Universal test and treat (UTT).

[50] Hayes RJ, Moulton LH. Cluster Randomised Trials: Taylor & Francis; 2009.

[51] Doherty T, Chopra M, Nsibande D, Mngoma D (2009). Improving the coverage of the PMTCT programme through a participatory quality improvement intervention in South Africa. BMC Public Health.

[52] Mate KS, Ngidi WH, Reddy J, Mphatswe W, Rollins N, Barker P (2013). A case report of evaluating a large-scale health systems improvement project in an uncontrolled setting: a quality improvement initiative in KwaZulu-Natal. South Africa BMJ Qual Saf.

[53] Youngleson MS, Nkurunziza P, Jennings K, Arendse J, Mate KS, Barker P (2010). Improving a mother to child HIV transmission programme through health system redesign: quality improvement, protocol adjustment and resource addition. PLoS One.

[54] Ovretveit J (2011). Understanding the conditions for improvement: research to discover which context influences affect improvement success. BMJ quality & safety..

[55] Taylor MJ, McNicholas C, Nicolay C, Darzi A, Bell D, Reed JE (2014). Systematic review of the application of the plan-do-study-act method to improve quality in healthcare. BMJ quality & safety.

[56] Bergstrom A, Skeen S, Duc DM, Blandon EZ, Estabrooks C, Gustavsson P (2015). Health system context and implementation of evidence-based practices-development and validation of the Context Assessment for Community Health (COACH) tool for low- and middle-income settings. Implementation science : IS.

