# Correction to: Addressing challenges in scaling up TB and HIV treatment integration in rural primary healthcare clinics in South Africa (SUTHI): a cluster randomized controlled trial protocol

**DOI:** 10.1186/s13012-019-0915-1

**Published:** 2019-06-13

**Authors:** Kogieleum Naidoo, Santhanalakshmi Gengiah, Nonhlanhla Yende-Zuma, Nesri Padayatchi, Pierre Barker, Andrew Nunn, Priashni Subrayen, Salim S. Abdool Karim

**Affiliations:** 1 Centre for the AIDS Programme of Research in South Africa (CAPRISA), Nelson R Mandela School of Medicine, Private Bag X7, Congella, Durban, 4013 South Africa; 2 CAPRISA-MRC TB-HIV Pathogenesis and Treatment Research Unit, Durban, South Africa; 3 Institute for Healthcare Improvement, Cambridge, MA USA; 4 Gillings School of Global Public Health, UNC Chapel Hill, Chapel Hill, USA; 5 Medical Research Council Clinical Trials Unit at University College London, London, UK; 6 BroadReach, Durban, South Africa; 7 Department of Epidemiology, Mailman School of Public Health, Columbia University, New York, NY USA

**Correction to: Implement Sci**

**https://doi.org/10.1186/s13012-017-0661-1**

Following publication of the original article [1], the authors reported an error in the wording of the study hypothesis. The error occurs in the Aims and Objectives sub-section of the Methods section and is highlighted in bold below.

The original article reads:

The hypothesis is that survival rates will be **lower** in TB, HIV, and TB-HIV co-infected patients accessing health care at clinics implementing the study intervention to deliver integrated TB-HIV care, compared to survival in patients accessing health care at clinics that provide only the standard of care for people with TB and or HIV.

The sentence should read:

The hypothesis is that survival rates will be **higher** in TB, HIV, and TB-HIV co-infected patients accessing health care at clinics implementing the study intervention to deliver integrated TB-HIV care, compared to survival in patients accessing health care at clinics that provide only the standard of care for people with TB and or HIV.
